# Differential Changes of Aorta and Carotid Vasodilation in Type 2 Diabetic GK and OLETF Rats: Paradoxical Roles of Hyperglycemia and Insulin

**DOI:** 10.1155/2012/429020

**Published:** 2011-10-01

**Authors:** Mei-Fang Zhong, Wei-Li Shen, Masaki Tabuchi, Kyoko Nakamura, Yi-Chen Chen, Cong-Zhen Qiao, Jin He, Jie Yang, Chuan Zhang, Zdravko Kamenov, Hideaki Higashino, Hong Chen

**Affiliations:** ^1^Department of Pharmacology and Shanghai Key Laboratory of Vascular Biology, Ruijin Hospital, Shanghai Jiao Tong University School of Medicine, 280 South Chong Qing Road, Shanghai 200025, China; ^2^Department of Pharmacology, Kinki University School of Medicine, Osaka 589-8511, Japan; ^3^Division of New Drug Research, School of Pharmacy, Second Military Medical University, Shanghai 200433, China; ^4^Clinic of Endocrinology, Medical University-Sofia, 1431 Sofia, Bulgaria

## Abstract

We investigated large vessel function in lean Goto-Kakizaki diabetic rats (GK) and Otsuka Long-Evans Tokushima Fatty diabetic rats (OLETF) with possible roles of hyperglycemia/hyperosmolarity and insulin. Both young and old GK showed marked hyperglycemia with normal insulin level and well-preserved endothelium-dependent and endothelium-independent vasodilation in aorta and carotid artery. There were significant elevations in endothelial/inducible nitric oxide synthase (eNOS/iNOS) and inducible/constitutive heme oxygenase (HO-1/HO-2) in GK. The endothelium-dependent vasodilation in GK was inhibited partly by NOS blockade and completely by simultaneous blocking of HO and NOS. In contrast, OLETF showed hyperinsulinemia and mild hyperglycemia but significant endothelium dysfunction beginning at early ages with concomitantly reduced eNOS. Insulin injection corrected hyperglycemia in GK but induced endothelium dysfunction and intima hyperplasia. Hyperglycemia/hyperosmolarity *in vitro* enhanced vessel eNOS/HO. We suggest that hyperinsulinemia plays a role in endothelium dysfunction in obese diabetic OLETF, while hyperglycemia/hyperosmolarity-induced eNOS/HO upregulation participates in the adaptation of endothelium function in lean diabetic GK.

## 1. Introduction

It is well established that type 2 diabetes is associated with increased macro- and microvascular complications. Hyperglycemia, the main characteristic of both type 1 and type 2 diabetes, is generally considered a detrimental factor in vascular dysfunction and therefore, diabetic patient treatment has focused primarily on tight blood glucose control. However, recent evidence from the most relevant clinical trials aiming at improving cardiovascular outcomes with intensive glycemic control does not support the assumption that strict glucose control exclusively is sufficient to reduce cardiovascular risk in complicated type 2 diabetes, questioning the role of hyperglycemia in poor cardiovascular outcomes [1–3]. 

Beside the debate in clinical settings, there are also controversies over the role of hyperglycemia in endothelium dysfunction and cardiovascular tolerance to ischemia in diabetic animals [4–9]. We have found that streptozotocin-induced type 1 diabetic rats demonstrated well-preserved coronary flow reservation after myocardial ischemia *in vitro* as well as unchanged or even slightly enhanced endothelium-dependent vasodilation in isolated aorta [6, 7]. We also found that hyperglycemia/hyperosmolarity could induce endogenous antioxidants including heme oxygenase (HO) in cardiovascular system that may contribute to the preserved vessel function in diabetes with severe hyperglycemia [6, 7, 10, 11]. There are other investigations showing unaltered or augmented endothelium-dependent relaxation at early stages of type 1 and type 2 diabetic rats [12–15], indicating different mechanisms involved in the two types of diabetes. 

Goto-Kakizaki rats (GK) are a highly inbred strain of glucose-intolerant Wistar rats that spontaneously develop type 2 diabetes without obesity [16]. Another kind of type 2 diabetic rats, Otsuka Long-Evans Tokushima Fatty rats (OLETF), are a strain of rats that develop hyperglycemia gradually after birth with obesity [17], resembling human type 2 diabetes with obesity. Our previous studies revealed decreased coronary flow after ischemia [18] and basal endothelium dysfunction [10] in 6- to 12-month-old OLETF. 

However, endothelium function may vary depending on different site of vascular beds, various stages, and severity of diabetes. And there is little investigation comparing large vessel function between diabetic rats of lean GK and obese OLETF at different stages. Therefore, we designed to compare vasodilation changes of aorta and carotid artery in diabetic lean GK and obese OLETF at early and later stages of diabetes. We also checked the effect of metabolic control on vessel function and intima with insulin injection in GK that had higher glucose but relatively normal insulin levels. The possible roles of NOS and HO in the endothelium function as well as the effects of hyperglycemia/hyperosmolarity on vessel eNOS/HO-1 were investigated. 

## 2. Methods

### 2.1. Animals

All experimental procedures were performed under protocols approved by the Animal Care Committee of the Animal Center at the Chinese Academy of Sciences in Shanghai and by the Committee on the Care and Use of Animals in Research at Shanghai Jiao Tong University/Kinki University School of Medicine in Japan.

Male GK and normal control Wistar rats at 3, 6, and 12 months of age were obtained from the Shanghai Laboratory Animal Center, Chinese Academy of Science. Male OLETF and nondiabetic control Long-Evans Tokushima Otsuka rats (LETO) at 2 months of age were supplied by the Tokushima Research Institute (Otsuka Pharmaceutical, Tokushima, Japan) and raised in the animal center of Kinki University School of Medicine, up to the same ages of GK until sacrifice. The animals were housed in two per cage in a temperature-controlled room with a 12 : 12-h light-dark cycle and had water and rat chow ad libitum. 

### 2.2. Blood Pressure Measurement

Systolic blood pressure and heart rate were measured in conscious state with tail-cuff method using a blood pressure analyzer (BP-98A, Softron, Beijing, China) before vessel tension experiment. 

### 2.3. Biochemical Determinations

Blood samples were collected for biochemical determination at the time of sacrifice. Blood lipids were measured using a biochemistry automatic analyzer (Hitachi, Tokyo, Japan). Serum insulin was determined with a rat radioimmunoassay insulin kit (Linco Research, St. Charles, USA). 

### 2.4. Assessment of Vessel Function

The method of vascular tension recording was modified slightly from our previous description [7, 10]. Briefly, after anesthesia, the thoracic aorta and common carotid artery were dissected carefully, cleaned of fat and adherent connective tissues, cut into segments of 2 to 3 mm (aorta) and 1 to 2 mm (carotid) in length, and mounted on two stainless-steel stirrups in 10 mL organ chambers with Krebs'-Henseleit buffer (KHB) containing (in mmol/L): NaCl 118.0, KCl 4.7, CaCl_2_ 2.5, MgSO_4_ 1.2, KH_2_PO_4_ 1.2, NaHCO_3_ 25.0, glucose 11.0, and Na_2_-EDTA 0.5, at 37°C. One stirrup was connected to an isometric force transducer for tension measurement in organ bath (ALC-B10 organ bath system, Alcott Biotech, Shanghai, China) and recorded with a data-acquisition system (MPA 2000, Alcott Biotech). 

The artery rings were stretched to a resting tension of 2.0 g (aorta) and 1.0 g (carotid), respectively, and allowed to equilibrate for 40 to 50 min, with the buffer changed every 10 to 15 min. The contraction response was evaluated twice with 60 mmol/L of KCl obtained by substituting an equimolar amount of KCl for NaCl in KHB. Endothelium function was assessed by 1 *μ*mol/L acetylcholine (ACh) when a contractile plateau was reached after 1 *μ*mol/L phenylephrine. Then, dose-response contraction to cumulative phenylephrine and dilation to acetylcholine (ACh)/sodium nitroprusside (SNP) were examined, increasing agent concentration at every 3 minute. 

To investigate possible mechanisms responsible for endothelium-dependent vasodilation, the rings were further incubated with NOS inhibitor *N* ^*ω*^-nitro-L-arginine methyl ester (L-NAME; 100 *μ*mol/L, 30 min) and HO inhibitor protoporphyrin IX zinc (II) (ZnPPIX; 2 *μ*mol/L, 30 min). ACh-induced dilation was reexamined after NOS and HO blockade. 

### 2.5. Immunofluorescence Study

Aortic and carotid segments of GK or OLETF with their respective controls at 12 months of age were immersed in 10% polyformaldehyde, embedded in paraffin, and sectioned for immunofluorescence colocalization of eNOS/iNOS and HO-1/HO-2. For double immunofluorescence reactions, sections were incubated in a mixture of primary antibodies including a monoclonal anti-eNOS antibody (1 : 1000 dilution; Sigma, St. Louis, MO, USA) and a polyclonal anti-iNOS antibody (1 : 100 dilution; Santa Cruz Biotechnology, Santa Cruz, CA, USA), or in a mixture of primary antibodies including a monoclonal anti-HO-1 antibody (1 : 100 dilution; Stressgen, Victoria, BC, Canada) and a monoclonal anti-HO-2 antibody (1 : 400 dilution; Assay Designs, Ann Arbor, MI, USA) [10, 19]. After incubation with the primary antibodies overnight at 4°C, sections were rinsed three times, followed by incubation with streptavidin-fluorescein isothiocyanate-conjugated and tetramethylrhodamine isothiocyanate-conjugated secondary antibodies for 30 min in a light-protected humidified chamber. The sections were then washed with PBS, and fluorescent eNOS/iNOS or HO-1/HO-2 was observed under a confocal microscope (Leica TCS SP2 Microsystems, Heidelberg, Germany). 

Digital images were captured and imported into JPEG files, and the areas of respective red and green fluorescence intensities (scale units ranging from 0 to 255, absolute red or green to absolute black) of intima were analyzed individually using Image-Pro Plus 5.0 software (Media Cybernetics, Bethesda, MD, USA). The obtained mean intensity for each image was inverted, with values of brighter color being higher, and divided by the respective area. The mean values in different groups were then compared with respective control values as reference [19]. 

### 2.6. Insulin Administration

One group of GK at 12 months were injected with protamine zinc insulin from 20 to 25 IU/kg/d subcutaneously for metabolic correction for 20 d before *in vitro* vessel function and morphology examination of aorta and carotid with transmission electron microscopy (TEM) as described before [10]. Blood glucose level was tested every other day and 5 to 6 days were necessary for achieving a relatively stable metabolic control. Therefore, the total insulin administration duration was from 25 to 26 days before vessel examination.

### 2.7. Measurement of Vessel Nitric Oxide (NO) and Reactive Oxygen Species (ROS)

Aortic tissues of Wistar rats, GK, and insulin-injected GK of 12 months were used for *in vitro* measurement of NO and ROS production. To measure intracellular NO production, the membrane-permeable fluorescent indicator 4-amino-5-methylamino-2′, 7′-difluorofluorescein diacetate (DAF-FM), was used. Briefly, aortic rings were loaded with 5 *μ*mol/L DAF-FM for 30 min at 37°C and fluorescence was measured at an excitation wave length of 485 nm and an emission wavelength of 538 nm every 1 min for 30 min [20]. Intracellular levels of ROS production were determined by measuring oxidative conversion of cell permeable 2′, 7′-dichlorofluorescein diacetate into fluorescent dichlorofluorescein, which was detected by fluorospectrophotometer at an excitation wavelength of 488 nm and an emission wave length of 535 nm [21]. 

### 2.8. Hyperglycemia/Hyperosmolarity Incubation and Western Blotting

Aorta rings from normal Wistar rats were incubated *in vitro* with hyperglycemia/hyperosmolarity by adding extra glucose or mannitol (50 mmol/L) into normal KHB with a final osmolarity of 350 mOsm/L [10]. After 5 h incubation with hyperglycemia/hyperosmolarity, the vessel rings were homogenized in lysis buffer (50 mmol/L *β*-glycerophosphate, 100 mmol/L Na_3_VO_4_, 2 mmol/L MgCl_2_, 1 mmol/L EGTA, 0.5% Triton X-100, and 1 mmol/L DTT) containing protease inhibitors (20 mmol/L pepstatin, 20 mmol/L leupeptin, 1,000 U/mL aprotinin, and 1 mmol/L phenylmethylsulfonyl fluoride). The soluble lysates were subjected to 10% (w/v) SDS-PAGE; proteins were then transferred to nitrocellulose membranes and blocked with 5% (w/v) nonfat milk/Tris-buffered saline Tween 20 (TBST) for 1 h at room temperature. Membranes were incubated overnight at 4°C with primary antibodies against tubulin (1 : 5000; Sigma), eNOS (1 : 1000; Sigma), or HO-1 (1 : 1000; Stressgen) in 5% (w/v) milk/TBST [10, 19]. The membranes were washed three times with TBST and then incubated with corresponding horseradish peroxidase-conjugated secondary antibody for 1 h, and immunoreactive bands were visualized using chemoluminescence. The relative protein levels were semiquantified with scanning densitometry and normalized against tubulin. 

### 2.9. Statistical Analysis

Data are presented as mean ± SEM. Differences in body weight, blood pressure, glucose, insulin, and other biochemical parameters among groups were analyzed with ANOVA followed by Student-Newman-Keuls post hoc analysis. Repeated measurement ANOVA was used for concentration-response relation. Significance was defined as *P* < 0.05.

## 3. Results

### 3.1. Basic Data

**Figure 1:**
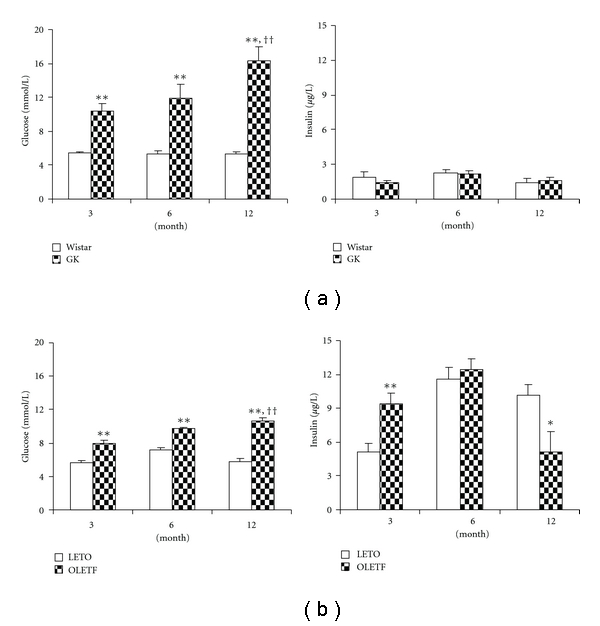
Blood glucose and insulin levels in Goto-Kakizaki rats (GK) and Otsuka Long-Evans Tokushima Fatty rats (OLETF). LETO: Long-Evans Tokushima Otsuka rats. Wistar is the control for GK, and LETO is the control for OLETF. Data are mean ± SEM, *n* = 8. *P < 0.05, **P < 0.01 versus respective controls, ^††^ *P* < 0.01 versus respective GK or OLETF at 3 months.

As shown in Figure 1, both GK and OLETF had significantly elevated glucose, with the level of GK higher than that of OLETF. The insulin levels in GK were identical to their normal control at 3 and 12 months, whereas OLETF had early increase in insulin which was maintained until middle age and declined at 12 months as compared with control LETO. Higher LDL-cholesterol and triglyceride levels were present in OLETF. However, GK showed higher HDL-cholesterol without elevation of LDL-cholesterol and triglyceride. The other basic data of GK and OLETF are shown in Tables 1 and 2. Besides normal blood pressure, GK showed increased heart rate at early and later stages. 

### 3.2. Vessel Relaxation

**Figure 2:**
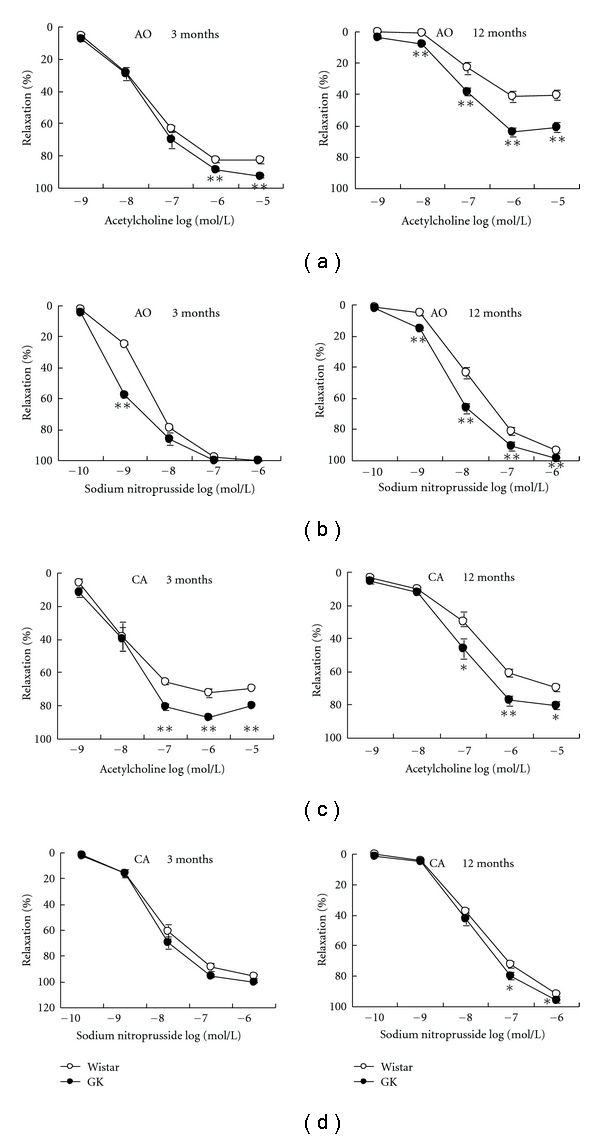
Endothelium-dependent and endothelium-independent vasodilation of the aorta (AO) and carotid artery (CA) in Goto-Kakizaki rats (GK) and age-matched control Wistar rats. Data are mean ± SEM, *n* = 16–24 segments. *P < 0.05, **P < 0.01 versus respective controls.

**Figure 3:**
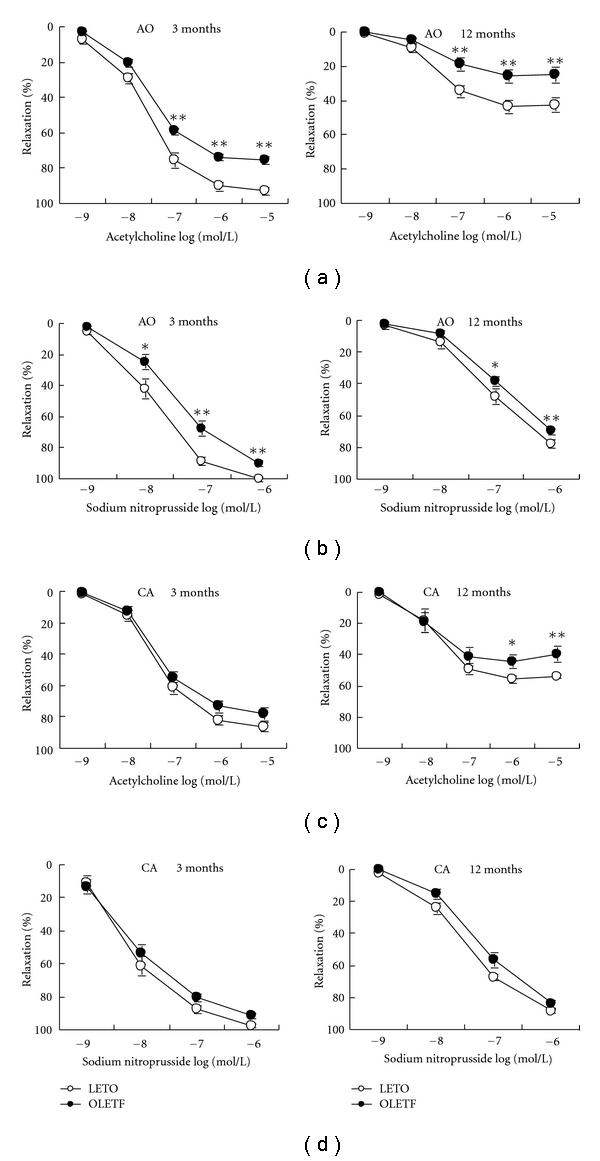
Endothelium-dependent and endothelium-independent vasodilation of the aorta (AO) and carotid artery (CA) in Otsuka Long-Evans Tokushima Fatty rats (OLETF) and age-matched control Long-Evans Tokushima Otsuka rats (LETO). Data are mean ± SEM, *n* = 10–14 segments. *P < 0.05, **P < 0.01 versus respective controls.

In GK at 3 and 12 months, both ACh- and SNP-induced dilation was significantly enhanced in aorta and carotid artery (Figure 2). The OLETF vessels, on the contrary, had significant impairment in ACh- and SNP-induced aortic dilation at 3 and 12 months with endothelium dysfunction of carotid artery at later stage (Figure 3).

**Figure 4:**
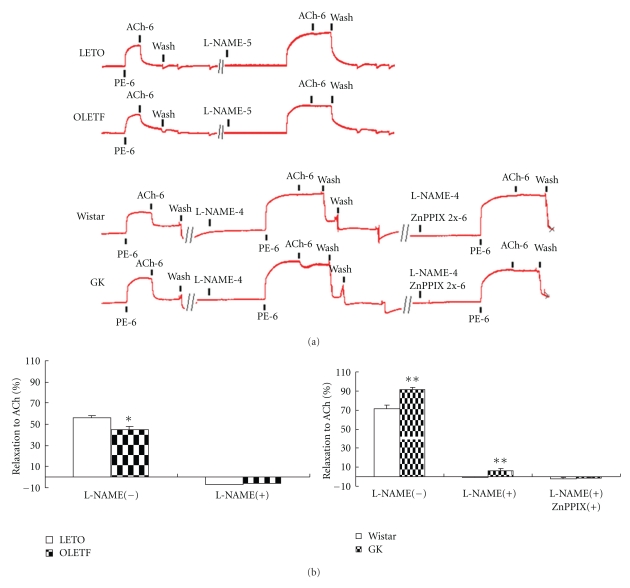
Aorta responses to acetylcholine (ACh) and phenylephrine (PE) in the presence of nitric oxide synthase blocker, *N* ^*ω*^-nitro-L-arginine methyl ester (L-NAME) or heme oxygenase blocker, protoporphyrin IX Zinc (II) (ZnPPIX) in OLETF: Otsuka Long-Evans Tokushima Fatty rat; LETO: Long-Evans Tokushima Otsuka rat; GK: Goto-Kakizaki rat. (a) representative tracings of vessel tension in 3-month-old OLETF and 12-month-old GK, respectively. (b) Relaxation of 12-month-old rat aorta rings to 1 *μ*mol/L ACh before and after NOS and/or HO blockade. Data are mean ± SEM, *n* = 10–12 segments. **P < 0.01 versus respective controls.

As demonstrated in Figure 4, NOS blocker L-NAME (100 *μ*mol/L) could abolish ACh-induced aortic dilation in normal control but ACh-induced dilation in GK remained about 6% after NOS inhibition. This non-NO-dependent dilation could not be abrogated by indomethacin but could be abrogated by HO blockade with ZnPPIX (2 *μ*mol/L) in the presence of L-NAME (100 *μ*mol/L), indicating NOS- and HO-related vasodilation. Carotid dilation induced by ACh could be abrogated completely by NOS blockade in both GK and OLETF, demonstrating NOS-related vasodilation in carotid artery without HO participation.

### 3.3. Vessel NOS and HO

**Figure 5:**
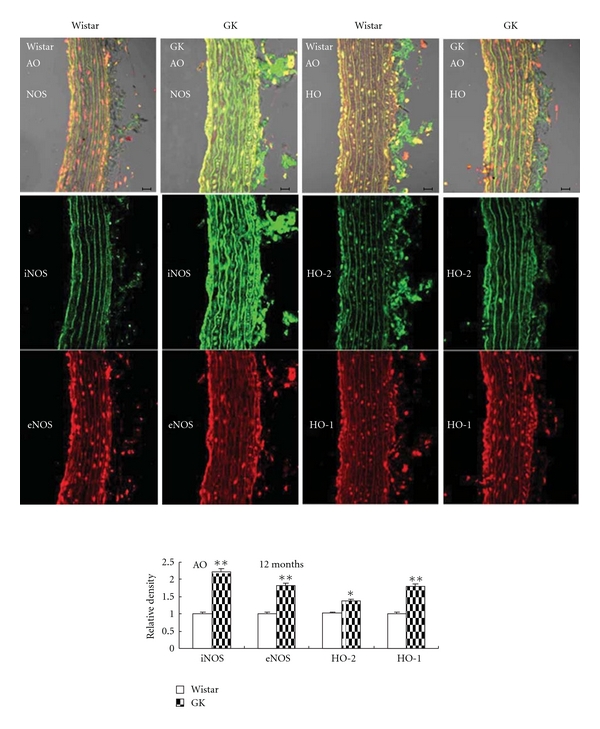
Immunofluorescent colocalization of endothelial nitric oxide synthase (eNOS, red) and inducible NOS (iNOS, green), constitutive heme oxygenase (HO-2, green) and inducible HO (HO-1, red) in aorta (AO) from control Wistar and Goto-Kakizaki rats (GK). The endothelial layer is on the left side and perivascular layers on the right side. Bar = 20 *μ*m. Data are presented as mean ± SEM, *n* = 6-7. *P < 0.05, **P < 0.01 versus respective controls.

**Figure 6:**
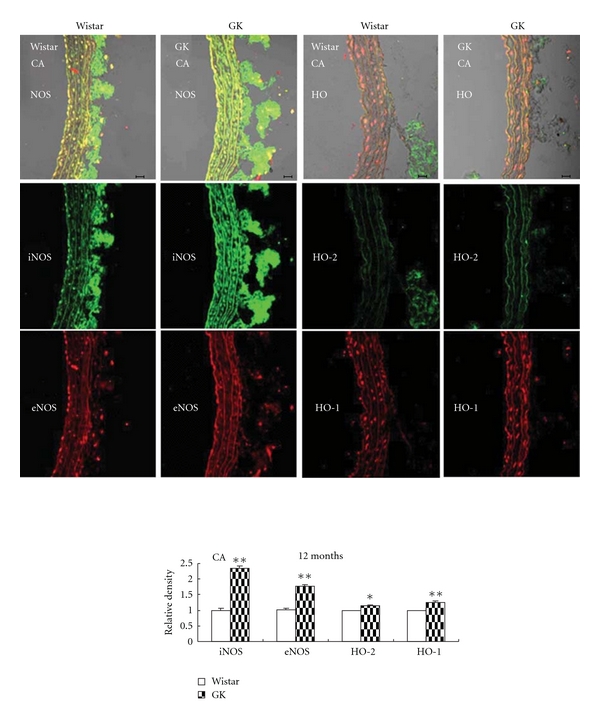
Immunofluorescent colocalization of endothelial nitric oxide synthase (eNOS, red) and inducible NOS (iNOS, green), constitutive heme oxygenase (HO-2, green) and inducible HO (HO-1, red) in carotid artery (CA) from control Wistar and Goto-Kakizaki rats (GK). Bar = 20 *μ*m. Data are presented as mean ± SEM, *n* = 6-7. **P < 0.01 versus respective controls.

Immunofluorescence examination demonstrated increased protein expression of aortic eNOS/iNOS and HO-1/HO-2 in GK, especially in the endothelial layer (Figure 5). In carotid artery, however, only NOS content was elevated and HO remained unchanged (Figure 6). 

**Figure 7:**
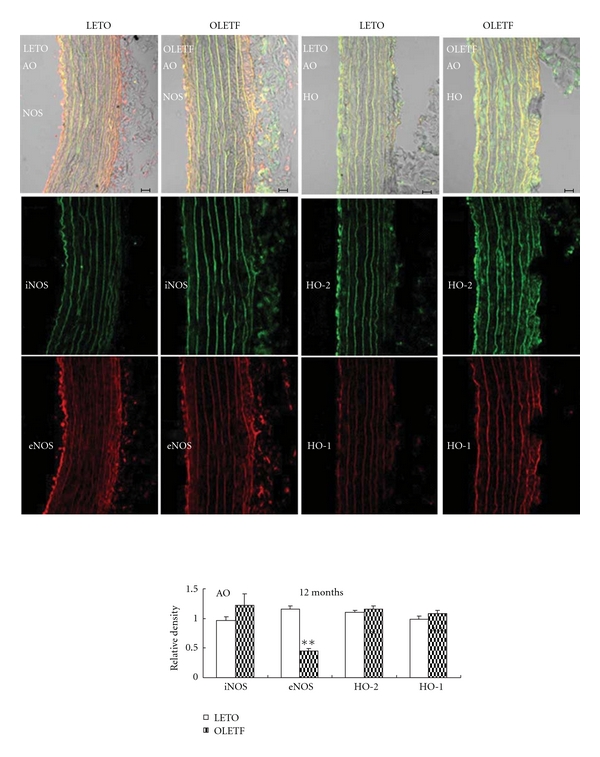
Immunofluorescent colocalization of endothelial nitric oxide synthase (eNOS, red) and inducible NOS (iNOS, green), constitutive heme oxygenase (HO-2, green) and inducible HO (HO-1, red) in aorta (AO) from control Long-Evans Tokushima Otsuka rats (LETO) and Otsuka Long-Evans Tokushima Fatty rats (OLETF). Bar = 20 *μ*m. Data are presented as mean ± SEM, *n* = 6-7. **P < 0.01 versus respective controls.

**Figure 8:**
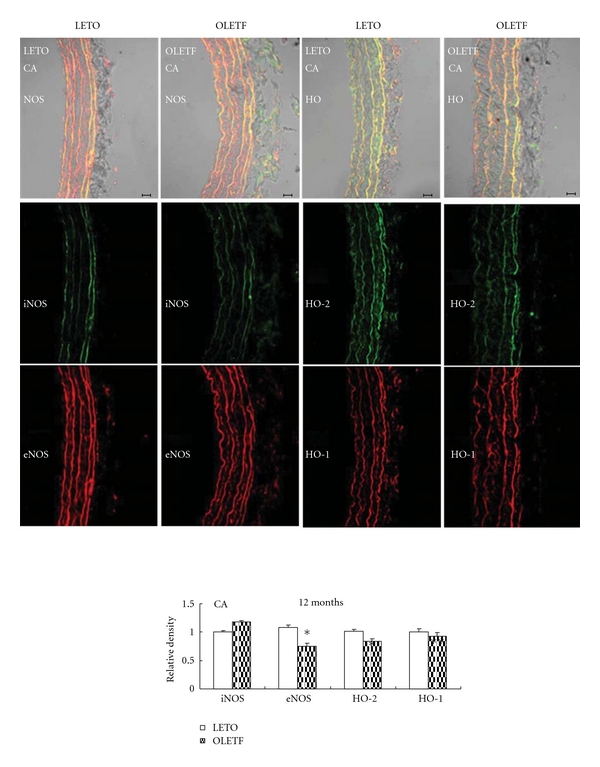
Immunofluorescent colocalization of endothelial nitric oxide synthase (eNOS, red) and inducible NOS (iNOS, green), constitutive heme oxygenase (HO-2, green) and inducible HO (HO-1, red) in carotid artery (CA) from control Long-Evans Tokushima Otsuka rats (LETO) and Otsuka Long-Evans Tokushima Fatty rats (OLETF). Bar = 20 *μ*m. Data are presented as mean ± SEM, *n* = 6-7. *P < 0.05 versus respective controls.

Both the aorta and carotid artery of OLETF exhibited decreased eNOS protein expression (Figures 7 and 8), suggesting impaired NOS system responsible for endothelium dysfunction in large vessels of the obese diabetic rats.

### 3.4. Vasodilation and Intima Changes after Insulin Administration

Daily insulin dose was adjusted around 20 to 25 IU/kg/d to maintain the blood glucose of GK at the range of 4 to 6 mmol/L for 20 days, with one GK out of eight died of hypoglycemia. Insulin injection in GK resulted in a marked reduction in blood glucose and elevation in serum insulin. The body weight, levels of glucose and insulin of GK injected with protamine zinc insulin at sacrifice were 422 ± 10 g, 4.0 ± 1.9 mmol/L, and 18.5 ± 5.4 *μ*g/L (*n* = 7), versus 368 ± 7 g, 19.5 ± 0.8 mmol/L, and 3.3 ± 0.6 *μ*g/L in control GK (*n* = 8), respectively. The body weight, levels of glucose and insulin in control Wistar were 409 ± 8 g, 5.6 ± 0.4 mmol/L, and 4.2 ± 0.5 *μ*g/L (*n* = 8), respectively. 

**Figure 9:**
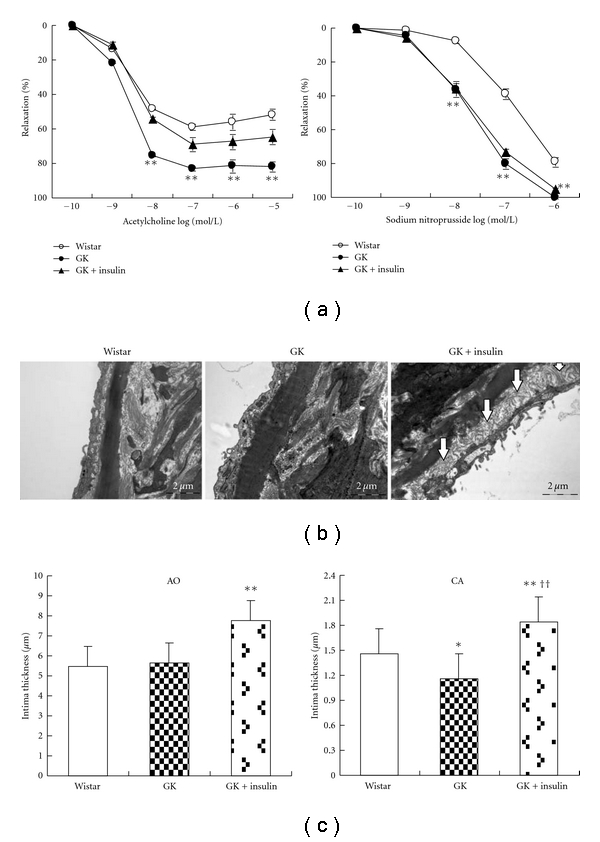
Vasodilation and intima morphology in aorta (AO) and carotid artery (CA) of 12-month-old Goto-Kakizaki rats (GK) after metabolic control with insulin injection. Arrows: subendothelial hyperplasia with many collagen fibers. Bar = 2 *μ*m. Data are presented as mean ± SEM, *n* = 13–18 segments. **P < 0.01 versus respective Wistar controls; ^††^ *P* < 0.01 versus GK.

Insulin administration reduced endothelium-dependent vasodilation with no change in endothelium-independent vasodilation (Figure 9(a)). Insulin also induced intima hyperplasia in both aorta and carotid artery. Subendothelial thickening with plenty of collagen fibers was the main characteristic of insulin-induced hyperplasia (Figures 9(b) and 9(c)).

### 3.5. Vessel Production of NO and ROS

Vessel production of NO was significantly increased in GK, which was reduced after insulin administration (relative NO production values of Wistar, GK, and GK+Insulin were 1.1 ± 0.2, 2.8 ± 0.5, 0.4 ± 0.2, resp., *n* = 4; GK versus Wistar and GK + Insulin, *P* < 0.01). The production of ROS in GK was also higher than control (relative ROS production values of Wistar, GK, and GK + Insulin were 1.2 ± 0.1, 6.5 ± 1.3, and 1.1 ± 0.2, resp., *n* = 4; GK versus Wistar, *P* < 0.01).

### 3.6. Hyperglycemia/Hyperosmolarity on Vessel eNOS/HO-1

**Figure 10:**
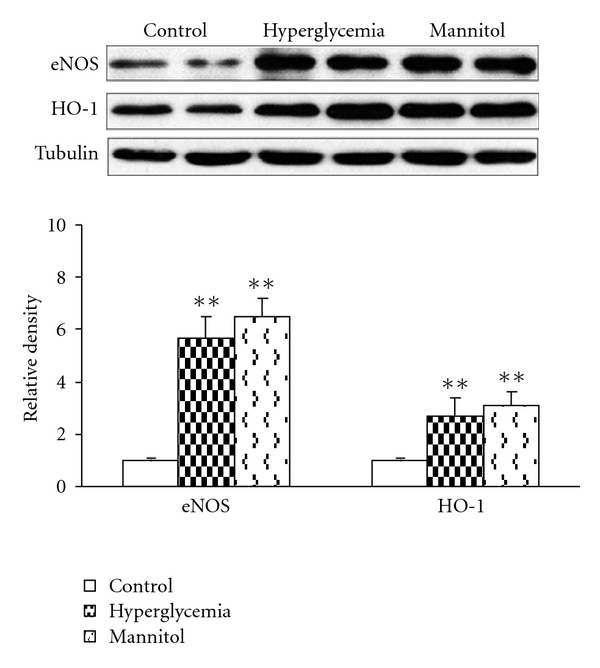
Protein expression of endothelial nitric oxide synthase (eNOS) and inducible heme oxygenase (HO-1) after hyperglycemia/hyperosmotic stimulation. Hyperosmolarity was made by adding extra glucose or mannitol (50 mmol/L) into Krebs-Henseleit Buffer with a final osmolarity of 350 mOsm/L (Krebs-Henseleit Buffer, 300 mOsm/L). Data are presented as mean ± SEM, *n* = 6. **P < 0.01 versus respective controls.

Protein expressions of eNOS and HO-1, both related to enhanced vasodilation, were upregulated in aorta after hyperglycemia/hyperosmolarity incubation for 5 h (Figure 10).

## 4. Discussion

The main findings in the present study are (1) the lean GK had marked hyperglycemia with well-preserved endothelium function at early and later stages, while the obese OLETF showed mild hyperglycemia but significant endothelium dysfunction and hyperinsulinemia at early stage; (2) the GK showed elevated vessel eNOS and HO protein and OLETF decreased vessel eNOS; (3) insulin injection corrected hyperglycemia in GK but induced endothelium dysfunction and intima hyperplasia; (4) hyperglycemia/hyperosmolarity *in vitro* upregulated vasodilating eNOS/HO-1.

 Endothelium dysfunction has been considered to contribute to the increased coronary artery disease in type 2 diabetic patients, and hyperglycemia is suggested to be one of the risk factors contributing to endothelium dysfunction. However, controversies exist on endothelium dysfunction in both type 1 and lean type 2 diabetic animals, especially in early stages [7, 10–15, 22–26]. The present findings in GK suggest that hyperglycemia *per se* seems not likely the cause for vessel dysfunction in diabetic rats at early or later stages. Rather, persistent hyperglycemia may be a preconditioning stimulus, inducing eNOS and HO that can exert both vasodilation as well as cardiovascular protection. Our results are supported by evidence from other groups showing that high glucose can induce eNOS elevation in GK aorta [15, 22] and in endothelial cells from human aorta [27].

Metabolic syndrome is highly prevalent in the modern society, with characteristics of central obesity, diabetes, hyperinsulinemia, hypertension, and dyslipidemia. Although the individual components of metabolic syndrome are clearly associated with increased coronary disease, there are also reports demonstrating that diabetes patients without metabolic syndrome have no greater prevalence of coronary artery disease [28, 29], indicating less importance of isolated hyperglycemia in macrovascular complications. In the present study, OLETF demonstrated typical metabolic syndrome that may be responsible for vessel dysfunction. It is noticeable that hyperinsulinemia was present at early stages of the 3-month-old OLETF, when endothelium dysfunction was already present. Other risk factors such as significantly higher LDL-cholesterol and triglyceride may contribute to the sustained endothelium dysfunction albeit the decline of insulin in the later stage of OLETF. Taking together the findings that OLETF with metabolic syndrome had mild hyperglycemia but early endothelium dysfunction, while GK without metabolic syndrome showed higher blood glucose but enhanced vasodilation, it is suggested that the conventional risk factors, such as obesity, hyperinsulinemia, and dyslipidemia, rather than hyperglycemia, contribute to the increased cardiovascular events in type 2 diabetes. 

In the 12-month-old GK aorta, we observed a non-NO-dependent acetylcholine-induced relaxation (ranging from 3% to 15%, with 6.4% in average) that could be abrogated by simultaneous inhibition of HO and NOS. This phenomenon could also be observed in type 1 diabetic model rats and was especially marked in GK with severe hyperglycemia [10, 11].

Heme oxygenase is a number of heat shock protein (hsp) family (also called hsp 32) that has antioxidant and cytoprotective properties. When breaking down heme, HO creates carbon monoxide (CO), another endogenous vasodilator that stimulates soluble guanylate cyclase in smooth muscle cells and regulates vascular tension in a manner similar to NO [30]. Our study found that exposure to hyperglycemia/hyperosmolarity could induce upregulation of eNOS and HO-1, supporting the hypothesis that in addition to NOS/NO system, HO/CO system may also contribute to the enhanced endothelium-dependent relaxation in GK.

The increased function of both NOS/NO and HO/CO systems may be a compensative response to hyperglycemia, which has been suggested as a harmful stimulus to various kinds of tissues and organs. In GK, hyperglycemia resulted in ROS and iNOS elevation and thus oxidative stress to endothelium. We have found that acute hyperglycemia (10 min) was detrimental to endothelium function with increased ROS production [31]; however, as the hyperglycemia stimulation sustained (5 h), it induced endogenous antioxidants including eNOS and HO [10]. Thus, hyperglycemia can be both a stress and a preconditioning factor that inducing endogenous protection. These findings may have some implications for the failure of clinical trials with antioxidants in diabetes, a way of treatment based on the rationale of oxidative stress-induced damage in diabetes [32]. The findings that hyperosmolarity stimulation was also effective on inducing eNOS/HO-1 as hyperglycemia suggests that hyperglycemia may act partly through hyperosmolarity in upregulating cardiovascular eNOS/HO-1 [6, 7]. However, further investigations are necessary to clarify the novel mechanisms of cardiovascular adaptation to hyperglycemia.

It is also interesting to find that metabolic correction with insulin reversed the enhanced endothelium function in GK, which may be due to the downregulation of eNOS/HO-1 as we found previously [10]. Insulin administration also resulted in intima hyperplasia, especially subendothelial thickening that may impede NO diffusion into smooth muscle to induce relaxation. The insulin-induced intima hyperplasia was also found in type 1 diabetes after balloon injury in rats [33] and may be responsible for accelerated coronary restenosis in human beings [34, 35]. Thus, our findings are consistent with the above reports, suggesting the metabolic correction by insulin could result in an unintentional hyperinsulinemia and intima hyperplasia that can be detrimental to endothelial function and vascular atherosclerosis. This might explain partly for the unfavorable or absence of favorable effects of intensive glucose control on cardiovascular outcomes in the ACCORD and ADVANCE or the Veterans Affairs Diabetes Trial (VADT), which use more insulin and insulin secretagogues frequently [1–3] as compared with the standard therapy. 

On the other hand, the Diabetes Control and Complications Trial/Epidemiology of Diabetes Interventions and Complications (DCCT/EDIC) studies have shown that in type 1 diabetic patients, intensive glucose control reduced the incidence of both micro- and macrovascular complications in the long-term during followup. The benefit was even present after the glucose returned to control level. Thus the prior glucose control exerted the benefit by way of “glucose memory” [36, 37]. The results from UKPDS also showed long-term benefits of a “memory” of intensive glucose control in macrovascular outcomes in type 2 diabetic patients [38, 39]. However, some assumptions have been put forward to explain the reasons for the apparent disparity, including the different targets of glucose control, weight gain, frequent hypoglycemia, different antihypertensive regiments, multiple risk factors, and the stages of diabetes when tight glucose control is initiated [2, 3, 36, 37, 40–42]. Compared with ACCORD and ADVANCE or VADT, DCCT/EDIC and UKPDS started glucose control at relatively early stages of diabetes with gradual achievement of target HbA1c, which were higher than that of ACCORD and ADVANCE or VADT at the end of the trials. The frequent use of insulin and secretagogues proved to be effective in rapid achieving of the target HbA1c in ACCORD but with more adverse events such as hypoglycemia. Our findings indicate that when lowering blood glucose, insulin administration abrogates the adaptive mechanisms of long-term severe hyperglycemia and promotes intima hyperplasia at the same time. The death from accidental hypoglycemia is possibly another reason of death during intensive blood control. Other risk factors, such as dyslipidemia and obesity in OLETF or weight gain in insulin injected GK, may also contribute to the endothelium dysfunction. Therefore, gradual glucose lowering combined with a more intensive approach to control the modifiable risk factors is necessary to minimize associated morbidity and mortality in type 2 diabetes. 

## 5. Conclusion

Both endothelium-dependent and smooth muscle-dependent vasodilation in aorta and carotid artery were enhanced in lean diabetic GK, but impaired in obese diabetic OLETF at early and later stages. Our data suggest that large vessels may adapt to sustained hyperglycemia/hyperosmolarity and developed a compensative way through upregulating eNOS/HO for vasodilation, while the clustering risk factors including marked obesity, hyperinsulinemia, and hypertriglyceridemia other than hyperglycemia are involved in vessel dysfunction of the obese OLETF. However, the exact effects of high glucose and insulin on vessel function and their mechanisms need further investigations.

## Figures and Tables

**Table 1:** Basic data of Wistar control and diabetic Goto-Kakizaki rats (GK).

	3 months		12 months	
	Wistar	GK	Wistar	GK
Body weight (BW), g	365 ± 8	307 ± 7**	498 ± 9	409 ± 10**
Systolic blood pressure, mmHg	115 ± 6	117 ± 7	118 ± 8	119 ± 5
Heart rate, beat/min	380 ± 8	439 ± 5**	382 ± 7	455 ± 12**
Total cholesterol, mmoL/L	1.82 ± 0.05	2.94 ± 0.11**	2.63 ± 0.13	3.11 ± 0.08**
LDL-cholesterol, mmoL/L	0.44 ± 0.02	0.40 ± 0.02	0.58 ± 0.07	0.55 ± 0.02
HDL-cholesterol, mmoL/L	1.13 ± 0.03	2.48 ± 0.09**	1.73 ± 0.08	2.49 ± 0.08**
Triglyceride, mmoL/L	1.19 ± 0.16	1.20 ± 0.14	1.86 ± 0.07	1.65 ± 0.13

*P < 0.05, **P < 0.01 versus respective age-matched Wistar controls. Data are mean ± SEM, *n* = 8.

**Table 2:** Basic data of nondiabetic control Long-Evans Tokushima Otsuka rats (LETO) and diabetic Otsuka Long-Evans Tokushima Fatty rats (OLETF).

	3 months		12 months	
	LETO	OLETF	LETO	OLETF
Body weight (BW), g	377 ± 6	478 ± 8**	678 ± 26	659 ± 32
Systolic blood pressure, mmHg	155 ± 12	145 ± 5	148 ± 5	150 ± 3
Heart rate, beat/min	406 ± 12	411 ± 16	358 ± 6	383 ± 14
Total cholesterol, mmoL/L	1.97 ± 0.06	2.35 ± 0.08**	3.13 ± 0.19	5.88 ± 0.64**
LDL-cholesterol, mmoL/L	0.13 ± 0.01	0.14 ± 0.01	0.25 ± 0.02	0.69 ± 0.12**
HDL-cholesterol, mmoL/L	0.77 ± 0.02	0.94 ± 0.03**	0.87 ± 0.04	1.43 ± 0.14**
Triglyceride, mmoL/L	0.24 ± 0.07	0.63 ± 0.06**	0.33 ± 0.04	2.03 ± 0.29**

*P < 0.05, **P < 0.01 versus respective age-matched LETO controls. Data are mean ± SEM, *n* = 8.
